# Genes involved in thoracic exoskeleton formation during the pupal-to-adult molt in a social insect model, *Apis mellifera*

**DOI:** 10.1186/1471-2164-14-576

**Published:** 2013-08-28

**Authors:** Michelle Prioli Miranda Soares, Angel Roberto Barchuk, Ana Carolina Quirino Simões, Alexandre dos Santos Cristino, Flávia Cristina de Paula Freitas, Luísa Lange Canhos, Márcia Maria Gentile Bitondi

**Affiliations:** 1 Departamento de Genética, Faculdade de Medicina de Ribeirão Preto, Universidade de São Paulo, Ribeirão Preto, SP, Brasil; 2 Departamento de Biologia Celular, Tecidual e do Desenvolvimento, Instituto de Ciências Biomédicas, Universidade Federal de Alfenas, Alfenas, MG, Brazil; 3 Centro de Engenharia, Modelagem e Ciências Sociais Aplicadas – CECS, Universidade Federal do ABC, Santo André, SP, Brazil; 4 The Queensland Brain Institute, The University of Queensland, Brisbane, Queensland, Australia; 5 Departamento de Biologia, Faculdade de Filosofia, Ciências e Letras de Ribeirão Preto, Universidade de São Paulo, Ribeirão Preto, SP, Brasil

**Keywords:** Cuticular protein genes, Metamorphosis, Molt, Thoracic musculature, Microarrays, Honeybee, *Apis mellifera*

## Abstract

**Background:**

The insect exoskeleton provides shape, waterproofing, and locomotion *via* attached somatic muscles. The exoskeleton is renewed during molting, a process regulated by ecdysteroid hormones. The holometabolous pupa transforms into an adult during the imaginal molt, when the epidermis synthe3sizes the definitive exoskeleton that then differentiates progressively. An important issue in insect development concerns how the exoskeletal regions are constructed to provide their morphological, physiological and mechanical functions. We used whole-genome oligonucleotide microarrays to screen for genes involved in exoskeletal formation in the honeybee thoracic dorsum. Our analysis included three sampling times during the pupal-to-adult molt, i.e., before, during and after the ecdysteroid-induced apolysis that triggers synthesis of the adult exoskeleton.

**Results:**

Gene ontology annotation based on orthologous relationships with *Drosophila melanogaster* genes placed the honeybee differentially expressed genes (DEGs) into distinct categories of Biological Process and Molecular Function, depending on developmental time, revealing the functional elements required for adult exoskeleton formation. Of the 1,253 unique DEGs, 547 were upregulated in the thoracic dorsum after apolysis, suggesting induction by the ecdysteroid pulse. The upregulated gene set included 20 of the 47 cuticular protein (CP) genes that were previously identified in the honeybee genome, and three novel putative CP genes that do not belong to a known CP family. *In situ* hybridization showed that two of the novel genes were abundantly expressed in the epidermis during adult exoskeleton formation, strongly implicating them as genuine CP genes. Conserved sequence motifs identified the CP genes as members of the CPR, Tweedle, Apidermin, CPF, CPLCP1 and Analogous-to-Peritrophins families. Furthermore, 28 of the 36 muscle-related DEGs were upregulated during the *de novo* formation of striated fibers attached to the exoskeleton. A search for *cis*-regulatory motifs in the 5′-untranslated region of the DEGs revealed potential binding sites for known transcription factors. Construction of a regulatory network showed that various upregulated CP- and muscle-related genes (15 and 21 genes, respectively) share common elements, suggesting co-regulation during thoracic exoskeleton formation.

**Conclusions:**

These findings help reveal molecular aspects of rigid thoracic exoskeleton formation during the ecdysteroid-coordinated pupal-to-adult molt in the honeybee.

## Background

Insect development occurs through a series of exoskeleton (cuticle) renewals, or molts, that are timed by ecdysteroids pulses. Molt succession includes apolysis (the separation of the cuticle from the epidermis), synthesis of a new cuticle and ecdysis (the shedding of the cuticle of the preceding instar, or stage).

After four molting episodes, the honeybee larva reaches the fifth larval instar without phenotypic changes, except for a considerable increase in size. The larva-to-pupa metamorphic molt takes place within the cuticle of the fifth larval instar. This is followed by pupal ecdysis, after which a white pupa breaks free from the larval cuticle. A genuine honeybee pupa exists for a relatively short period, lasting approximately 40 h from pupal ecdysis. Apolysis marks the onset of adult cuticle synthesis and deposition. During the next 160 h, the bee is a pharate-adult, meaning that it is producing the adult cuticle underneath the pupal cuticle. Melanization starts and intensifies in the adult cuticle throughout the final half of the pharate-adult period, even after adult ecdysis. Concomitantly, the adult cuticle becomes increasingly sclerotized [1-4]. Thus, development toward the adult stage involves the reconstruction and maturation of the definitive cuticular exoskeleton, formation of internal tissues and organs, and programmed cell death of many larval tissues.

The structure, chemical composition, mechanical properties and renewal of the insect exoskeleton during growth and metamorphosis have been extensively studied [5,6]. The exoskeleton mainly consists of the polysaccharide chitin and a variety of structural proteins, the CPs. Nearly a decade ago, Willis *et al*., [7] compiled 139 insect CPs derived from direct sequencing of purified proteins, or from conceptual translations of DNA sequences. Since then, insect genome projects have expanded the number and types of potential CPs, thus underscoring the variety of cuticle-forming proteins [8]. However, except for extensive studies on cuticular gene expression in *Anopheles gambiae*[9-11], few putative CP genes in annotated insect genomes have been experimentally validated (by determining transcript tissue-specificity or developmental profiles). In *A. mellifera*, only six of the 47 CP genes screened in the annotated genome [8] have been validated, including the *AmelCPR14* gene [12] (bearing the chitin-binding R&R Consensus [13]), three genes in the Apidermins class [14], and the Tweedle class genes, *AmelTwdl1* and *AmelTwdl2*[15].

Thoracic exoskeleton construction and differentiation in pharate-adults occur concomitantly with the *de novo* formation of striated muscle fibers. The larval thoracic muscles are entirely disintegrated during the honeybee metamorphic molt, and are replaced by imaginal muscles originating from myoblast precursors, which elongate, join, and form the striated muscle fibers. Motor function is accomplished by the attachment of these muscle fibers to specific points, or ingrowths, in the developing thoracic exoskeleton [1]. Thus, the integument (epidermis and cuticular exoskeleton) and associated musculature form a cohesive and functional structure.

The morphological and cellular changes in the dorsal portion of the honeybee thorax during the pupal-to-adult molt were previously characterized in our laboratory using conventional light microscopy [4] and transmission electron microscopy [16]. In addition to these detailed morphological descriptions, we used oligonucleotide microarray hybridization analysis, real-time RT-PCR (RT-qPCR) transcript quantification and fluorescent *in situ* hybridization to extend our knowledge. Unlike recent approaches in other insects, including microarray-based studies [17-19], we studied honeybee gene expression in the context of reconstruction of the hard exoskeleton that forms the thoracic dorsum, which includes the pronotum, mesonotum, metanotum, propodeal tergum (the dorsal part of the first abdominal segment that, in bees and wasps, becomes part of the adult thorax [1]) and the subjacent musculature. In contrast to the diversity of cuticle types that covers the entire insect body (hard/flexible; dark/clear), the thoracic dorsum is uniformly hardened and darkened. Thus, genes involved in the formation of a hard cuticle could be characterized. The thorax also has fewer glands that could contribute to the total RNA pool. Although there are integument glands in the thorax [20-22], they are much less abundant than in the abdomen. As described in *Drosophila*, the thoracic dorsum (notum) and associated musculature both originate from the same embryonic precursors (the wing imaginal discs) [23]. Thus we also investigated their potential shared gene regulatory networks for coordinated development.

Our microarray analyses identified genes involved in adult thoracic dorsum formation. We used this information to characterize potential binding sites for known transcription factors in the upstream control region (UCR) of the differentially expressed genes (DEGs). CP genes and muscle-related genes involved in thoracic dorsum formation were used to construct a putative gene regulatory network. Ecdysteroids control the pace of pupal-to-adult molt and adult thoracic dorsum development; therefore, we could also identify CP- and muscle-related genes that are potentially regulated by these hormones.

## Methods

### Africanized *Apis mellifera* samples

Newly-ecdysed pupae (white-eyed/unpigmented cuticle, Pw phase), pupae-in-apolysis (pink-eyed/unpigmented cuticle, Pp phase) and pharate-adults (brown-eyed/unpigmented cuticle, Pb phase; brown-eyed/light pigmented cuticle, Pbl phase) were collected from colonies maintained at an Experimental Apiary (University of São Paulo, Ribeirão Preto, SP, Brazil) and staged according to the criteria established by Michelette and Soares [3]. Dissections of the dorsal portion of the thorax, including pronotum, mesonotum, metanotum and the propodeal tergum, were performed in Ringer saline (0.17 M NaCl; 0.01 M KCl; 0.003 M CaCl_2_). The total RNA was extracted from samples using 1 mL Trizol (Invitrogen, Life Technologies, cat. 15596–026) following manufacturer’s instructions. The *A. mellifera* experiments performed in the present study comply with the current laws of Brazil.

### Semithin sections

The thoracic dorsum of pupae and pharate-adults were dissected and immediately fixed in a mixture of glutaraldehyde 2% and paraformaldehyde 2% in 0.1 M cacodylate buffer, pH 7.2 for 2 h at 4°C. Next, they were washed in 0.1 M phosphate-buffered saline, pH 7.2 (PBS) and re-fixed for 30 min in 1% osmium tetroxide in 0.1 M PBS. The samples were then dehydrated in a graded acetone series and propylene oxide, pre-embedded in LX112 resin (Leica) in propylene oxide (1:1) and, finally, embedded in pure LX112 resin. Semithin sections (0.5 μm) where stained with toluidine blue.

### Microarray hybridization and analysis

Oligonucleotide-based microarrays (BeeOligo121106) were purchased from the Keck Center for Comparative and Functional Genomics, University of Illinois, Urbana-Champaign, USA. The array contains oligonucleotide probes including 12,915 protein-coding gene predictions from the honeybee genome, and probes specific for ESTs, pathogens and parasites in a total of 14,400 oligos, comprising each 36 nucleotides double-spotted on glass microscope slides [http://www.ebi.ac.uk/aerep/result?queryFor=PhysicalArrayDesign&aAccession=A-MEXP-755].

The experiments were designed and performed to meet the Minimum Information About a Microarray Experiment (MIAME) specifications. The obtained microarray data were deposited at the Gene Expression Omnibus database [GEO:GSE43047]. The microarray experiments compared gene expression in the thoracic dorsum (including the pronotum, mesonotum, metanotum and propodeal tergum) of newly-ecdysed pupae, pupae-in-apolysis and pharate-adults (Pbl phase) collected at the same time from a single honeybee colony. Two separate pools of 10 thoraces (only the dorsal portion) were prepared for each of these developmental phases. Total RNA was extracted from the pooled samples using Trizol reagent and following manufacturer’s protocol. RNeasy Mini Kit, QIAGEN, cat. 74104, was used to purify the extracted RNA, which was quantified in Nanodrop (ND 1000). One microgram of the purified total RNA was used for RNA amplification with Amino AllylMessageAmp™ II aRNA Amplification Kit (Ambion), and following manufacturer´s protocol.

The microarray slides were UV cross-linked and subsequently incubated in 0.2% SDS for 2 min and rapidly in water and isopropanol, and centrifuged for 3 min at a low speed. Pre-hybridizations were performed in a mixture of 20 mL formamide, 33.2 mL SSC 20x, 10 mL Denhardt’s solution 50x, 0.5 mL tRNA 10 mg/mL, 1 mL SDS 10% and 34.4 mL Milli-Q water for 60 min at 42°C. The slides were then rinsed twice in Milli-Q water, soaked in isopropyl alcohol and dried by centrifugation at low speed for 3 min.

Twenty micrograms of the amplified RNA samples (aRNA) were used for dual channel microarray hybridization with Cy3 and Cy5 dyes (dye swap). Dye swaps were done for each comparison and two slides were used to evaluate the differential expression. The RNA was dye-coupled and combined with hybridization buffer (49% Formamide, 49% SSC 20x and 0.2% SDS), pre-heated at 55°C for 3 min, applied to arrays, and hybridized for 17 h at 42°C in single slide hybridization chambers placed in a water bath. A series of 30 s shaking washes in 2× SSC plus 0.1% SDS; 2× SSC; 0.1× SSC and Milli-Q water, at room temperature, removed probe excess. The hybridized slides were spin dried and scanned using an Axon Genepix 4000B scanner (Molecular Devices, Sunnyvale, CA) with GENEPIX software (Agilent Technologies, Santa Clara, CA), 10-micron resolution, Cy3 with Green Laser (532 nm), and Cy5 with Red Laser (635 nm). GenePix Pro 6.0 software was used for quantification of the spots and image analysis with default parameters.

The microarray data analyses were performed with Limma package (Biocondutor project [http://www.bioconductor.org/]) together with R [http://www.r-project.org/] [24]. The Normexp method was used for background normalization adding an offset of 50 to the corrected intensities. Normalization within-arrays was done using print-tip loess to adjust imbalances between the green and red dyes due to spot intensity and spatial position on the slide. Average intensities were normalized between-arrays using the Aquantile method. After normalization, the Log2 ratio of gene expression differences between the developmental phases was calculated. Fold-change values and standard errors were estimated by fitting linear regression to the normalized expression data. The empirical Bayes statistics (moderated t-statistics) was used for differential expression analysis [24]. The p-value of the differentially expressed genes was corrected using FDR (<0.05) [25].

The DEGs were annotated according to Gene Ontology (GO) terms for Biological Process (level 3), Molecular Function (level 2) and Cellular Component (level 3) [26] using DAVID (Database for Annotation, Visualization and Integrated Discovery) Gene Functional Classification Tool (version 6.7) [27].

### RT-qPCR analysis

The RNA samples were incubated in the presence of 3 units of RNase-free DNase (Promega) for 40 min at 37°C to eliminate contaminant DNA, and for 15 min at 70°C to inactivate the DNase. First-strand cDNA was synthesized by reverse transcription (RT) using 2.5 μg of total RNA, SuperScript II reverse transcriptase and the oligo(dT)_12–18_ primer (Invitrogen, Life Technologies). Reactions not including the SuperScript II reverse transcriptase, or cDNA template, were prepared as negative controls and analyzed in parallel. PCR amplification was carried out at a 7500 Real Time PCR System (Applied Biosystems) using 20 μL reaction volumes containing 10 μL SYBR Green Master Mix 2x (Applied Biosystems), 1 μL cDNA, 7.4 μL water, and 0.8 μL of each gene-specific primer (10 mM) (Additional file 1: Table S1). The PCR conditions were 50°C for 2 min and 95°C for 10 min followed by 40 cycles of 95°C for 15 s, and 60°C for 1 min.

When possible (because the CP genes are in general small and some contain only one intron), the primers used for amplification of the target genes were designed to span an intron, thereby serving as control for genomic DNA contamination. The gene encoding a ribosomal protein, *Amrp49*, which is expressed in similar levels during the pupal and pharate-adult stages of the honeybee, and was validated as being a suitable reference gene [28], was used for normalizing the RT-qPCR data. The primers for the *Amrp49* gene were designed to span one intron.

Each PCR run was followed by melt curve analysis. Primer specificity and absence of prime dimers were confirmed by the sharp melting curve of the PCR product and by the presence of a single band on agarose gel electrophoresis. The efficiency (E) of PCR amplification was calculated from the slope of standard curves (serial dilutions of the cDNA) using the equation E = 10^(−1/slope)^ and set near 2.

To check reproducibility, each SYBR green assay was done twice (technical replicate) and repeated with 3 independent samples (biological triplicate). The baseline and threshold were correctly set. The data quantification and normalization relied on the calculation of target threshold cycle (Ct) values and reference gene Ct values in qBase^PLUS^ version 2 software [29].

### cDNA cloning and sequencing

The cDNAs obtained from two putative CP genes, GB12449 and GB12811, were amplified using specific primers (Additional file 2: Table S2). PCR products were subjected to electrophoresis on agarose gel, purified with Perfectprep Gel Cleanup kit (Eppendorf), and cloned into pGEM-T Easy Vector (Promega). Insert-containing plasmids were sequenced using M13 forward and reverse universal primers. Dideoxy sequencing was performed in an ABI Prism 310 DNA Analyser using BigDye Terminator v3.0 Cycle Sequencing Ready Reaction (Applied Biosystems). Sequencher™software version 4.7 (Gene Codes Corporation) was used to analyze the sequences, which were then mapped to the annotated honeybee genome using Artemis software (Release 7, The Sanger Institute).

### Fluorescent *in situ* hybridization

*In situ* hybridization was carried out to investigate the presence of GB12449 and GB12811 transcripts in the honeybee epidermis. To construct the fluorescent probes, pairs of specific primers were designed to amplify regions of the GB12449 and GB12811 DNA sequences comprising 300 and 379 nucleotides, respectively (Additional file 2: Table S2). Single-stranded antisense and sense probes were synthesized using the FISH Tag RNA Green kit (Invitrogen, Life Technologies, cat. F32952), following manufacturer’s instructions.

Thoracic samples were dissected in cold Ringer saline and fixed in 1 mL heptane, 80 μL HEPES buffer (0.1 M HEPES, pH 6.9, 2 mM MgSO_4_, 1 mM EGTA), 100 μL 8% paraformaldehyde, 20 μL dimethyl sulfoxide (DMSO) for 30 min under shaking. The fixative was discarded, the samples were quickly rinsed in absolute methanol (2 rinses) and in absolute ethanol (2 rinses), and then stored up to 2 weeks in ethanol at −20°C or immediately rehydrated in PBS pH 7.4 containing 0.1% Tween-20 (PTw). After an additional fixation during 20 min in a 9:1 v/v mixture of 4% paraformaldehyde/0.1% Triton X-100 and DMSO, the samples were washed in PTw. To facilitate permeabilization and mRNA probe penetration, samples were incubated in a freshly prepared 20 μg/mL proteinase K in PTw for 5 min, followed by washes in a filter-sterilized 10 mg/mL glycine solution. Samples were then rinsed in PTw and re-fixed, as above. After washes in PTw, samples were equilibrated in hybridization solution (HS) (50% formamide, 4x standard saline citrate, 1x Denhardt’s solution, 250 μg/mL yeast total RNA, 250 μg/mL boiled salmon testes DNA, 50 μg/mL heparin, 0.1% Tween 20, 5% dextran sulfate), washed in 1:1 PTw/HS and subsequently in HS. Equilibrated samples were transferred to fresh tubes and pre-hybridized in HS for 1 h at 45°C. Sense and antisense probes were separately diluted in HS (200 ng/mL), heat-denatured for 2 min at 98°C, chilled on ice and added to the pre-hybridized samples. Hybridization was carried-out overnight at 45°C under gentle shaking. The hybridized samples were washed in HS: PTw (3:1, 1:1 and 1:3 v/v) and in PTw solution. For cell nuclei co-localization the thoracic samples were stained with diamidino-2-phenylindole (DAPI) diluted 1:4000 in PTw, followed by washes in PTw. The thoracic pieces were transferred to 70% glycerol in PTw, and mounted on slides using SlowFade ® Gold (Invitrogen) for observation under a confocal microscope TCS-SP5 AOBS (Leica).

### Characterization of potential regulatory sequences

We retrieved functional information from the Gene Ontology (GO) database [26] for the *D. melanogaster* ortholog genes differentially expressed in the honeybee thoracic dorsum during the pupal-to-adult molt. A pipeline for *cis*-regulatory motifs discovery was designed based on reliable strategies previously proposed by MacIsaac and Fraenkel [30], and adapted to analyze the honeybee genome [31,32]. This pipeline integrates three motif-detection programs: AlignAce [33], MEME [34] and MDscan [35]. Honeybee intergenic databases were constructed for 3 kb sequence sizes that were trimmed whenever another open reading frame (ORF) was found to be flanking these regions. These databases were exploited for score calculations using group specificity scores (Church scores) [36], ROC-AUC scores [37] and Enrichment scores [38]. Two additional specific score metrics, the MAP score from AlignAce and MDscan and the E-value from MEME, were also used as a first filter for selecting the most significant motifs (MAP > 5 and E-value ≤ 1e-05). The second filter was set up to decrease the amount of spurious hits among the identified DNA motifs (Church ≤ 1e-04, ROC-AUC ≥ 0.7 and P-value for enrichment ≤ 0.01). The main criterion for identifying known cis-regulatory sites among the overrepresented motifs was the alignment of the PSSM (Position-Specific Scoring Matrix) with the transcription factor binding sites as described in the TRANSFAC database, version 2010.1 [38]. Only the alignments passing a threshold of 70% identity for each PSSM were considered as significant matches. We used concepts of graph theory and complex networks [39].

We also used the consensus sequence for Fushi Tarazu Transcription Factor 1 (FTZ-F1) binding site (YCAAGGTCR; [40]) to build a PSSM and then identify putative FTZ-F1 binding sites (at least 70% similar to the consensus sequence) in the 3 kb upstream intergenic regions of selected DEGs. We used the same score metrics (Church score, ROC-AUC and Enrichment score) to estimate the specificity of FTZ-F1 binding sites.

## Results

### Ecdysteroid-coordinated development of the thoracic dorsum during the pupal-to-adult molt

**Figure 1:**
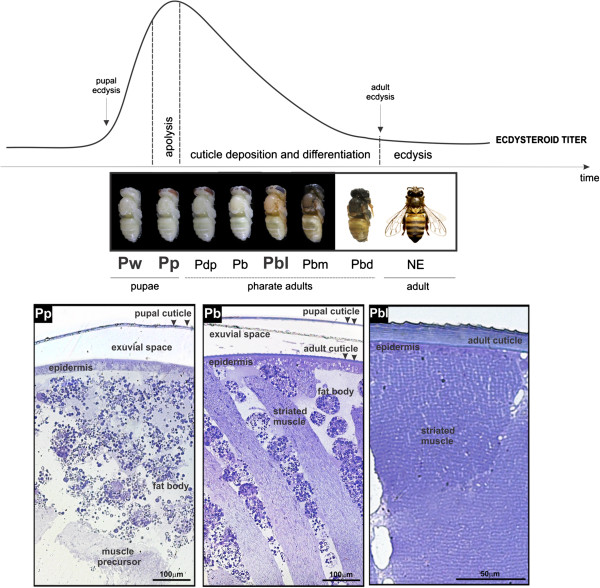
**Morphological changes in thoracic dorsum during molting.** Thoracic dorsum development in the honeybee during the ecdysteroid-regulated pupal-to-adult molt. Ecdysteroid titer modulation adapted from Pinto *et al.*[41]. Photos of bees in the sequential phases of the pupal-to-adult molt are shown. The abbreviations in bold under the photos represent the developmental phases used for gene expression analysis: Pw (newly-ecdysed-pupa), Pp (pupa-in-apolysis) and Pbl (pharate-adult). Apolysis is triggered in the Pp phase by the ecdysteroid peak. Deposition and differentiation of the adult exoskeleton occurs under declining ecdysteroid titer in the successive pharate-adult phases (Pdp, Pb, Pbl, Pbm and Pbd). NE: newly-emerged worker bee. Toluidine blue-stained semithin sections of the thoracic dorsum from pupae-in-apolysis (Pp phase), and pharate-adults (Pb and Pbl phases) are shown at the bottom. The detached pupal cuticle (arrowheads) is evident in pupae-in-apolysis (Pp phase) and pharate-adults (Pb phase). Muscle precursors and abundant fat body cells could be observed in pupae-in-apolysis (Pp phase). Adult cuticle deposition on epidermis (arrowheads), and differentiating muscle fibers interspersed with fat body cells were seen in early pharate-adults (Pb phase). The fat body cells disappear from the thoracic dorsum and muscle fibers contact epidermis in the next pharate-adult phase (Pbl).

Figure 1 shows the ecdysteroid titer-coordinated changes in external morphology (exoskeleton) during pupal-to-adult transition. Toluidine-stained cross sections of the thoracic dorsal integument and subjacent tissues are also shown for pupae-in-apolysis and two successive pharate-adult phases. The ecdysteroid peak induces the detachment of the pupal cuticle from the epidermis. The exuvial fluid is then secreted by the epidermis in the space created beneath the detached pupal cuticle. Next, the adult cuticle (shown in pharate-adults cross sections) is gradually deposited on the epidermis. The thoracic sections of the newly-ecdysed-pupae (not shown) and pupae-in-apolysis are histologically very similar, except that in the former the cuticle is still attached to the epidermis, while in the latter it is already detached.

### Changes in gene expression in the thoracic dorsum during pupal-to-adult development

The cDNA microarray analysis revealed 995 and 1,497 spots corresponding to up- or downregulated genes in the thoracic dorsum of pharate-adults compared with newly-ecdysed-pupae and pupae-in-apolysis, respectively. These genes represent 6.9% and 10.4%, respectively, of the 14,400 oligonucleotides spotted on the microarray slides. The oligonucleotide set included the following: Official Gene Set sequences (OGS); variable exons from the antimicrobial peptide apidaecin and from the IG-family gene Dscam; representative genes from viral, fungal, bacterial, and microsporidian pathogens of honeybees; and non-OGS expressed sequence tags (ESTs) from a subtractive library biased toward larval genes upregulated upon exposure to *Paenibacillus larvae*. Our analyses focused exclusively on the OGS sequences, which included 862 (86.63%) and 1,304 (87.11%) genes showing changes in their expression levels in pharate-adults compared with newly-ecdysed-pupae and pupae-in-apolysis, respectively. Some of these oligonucleotides were duplicated in the microarray slides; thus, there were 761 and 1,173 unique DEGs in the respective comparisons. Notably, statistically significant changes in gene expression were not detected during the newly-ecdysed-pupae to pupae-in-apolysis transition; the changes were greater, and therefore statistically significant, after apolysis.

**Figure 2:**
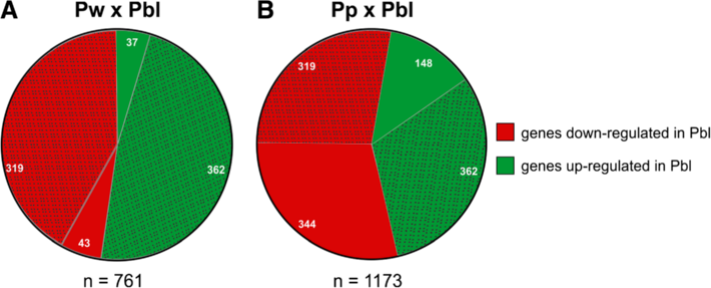
**Number of DEGs in the microarray analysis. (A)** Comparison between newly-ecdysed-pupae (Pw phase) and pharate-adults (Pbl phase). **(B)** Comparison between pupae-in-apolysis (Pp phase) and pharate-adults (Pbl). The dotted areas in the graphs represent the number of DEGs that are common to both comparisons.

The numbers of DEGs in the thoracic dorsum are shown in Figure 2. Some of these genes were differentially expressed in both comparisons; therefore, we compiled a total of 1,253 unique DEGs. These data highlighted the changes in gene expression during the transition from pupal-to-adult thoracic dorsum. The up- and downregulated genes are listed in Additional file 3: Table S3 and Additional file 4: Table S4.

**Figure 3:**
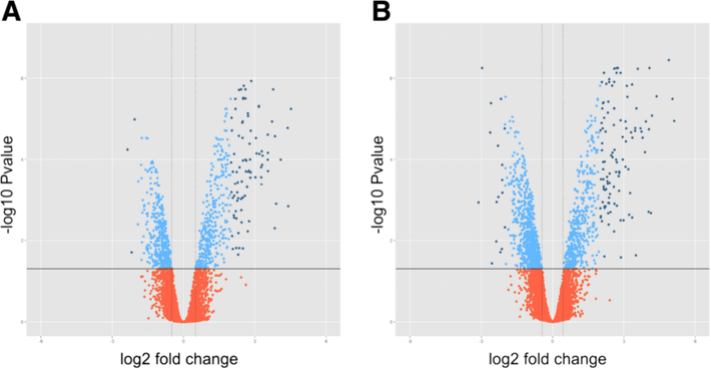
**Differential gene expression in the thoracic dorsum during the pupal-to-adult molt. (A)** Comparison between newly-ecdysed-pupae (Pw phase) and pharate-adults (Pbl phase). **(B)** Comparison between pupae-in-apolysis (Pp phase) and pharate-adults (Pbl phase). Plots of significance and fold-change are indicated in the y- and x-axes, respectively. The horizontal line indicate p value = 0.05. The genes that display statistically significant differential expression (p value < 0.05) and high magnitude fold change (Log2FC cutoff = 0.5) are represented as light- and dark-blue dots above the horizontal line (dark-blue dots display Log2 fold-change greater than 2). Red dots below the horizontal line indicate p value > 0.05 and small magnitude fold change.

Figure 3 illustrates the magnitude of the differences in gene expression between the pupal and pharate-adult thoracic dorsum.

### Gene ontology (GO) functional analysis

DAVID was used for GO analysis of DEGs that were orthologous to *D. melanogaster* genes. The comparisons of pharate-adults with newly-ecdysed-pupae or pupae-in-apolysis revealed 705 (92.64%) and 1,086 (92.58%) DEGs, respectively, with predicted orthology to *D. melanogaster* genes.

**Figure 4:**
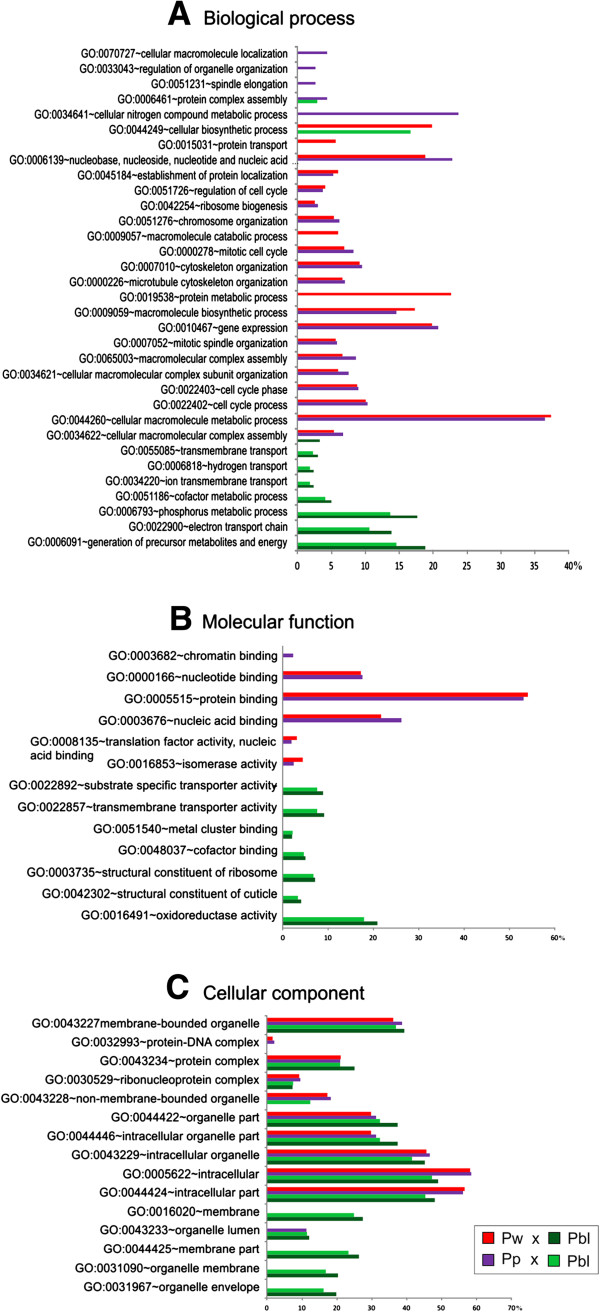
**Gene Ontology functional analysis.** GO functional analysis clustered the pupal and pharate-adult DEGs in distinct **(A)** Biological Process and **(B)** Molecular Function categories. **(C)** Several of the Cellular Component categories are shared by pupal and pharate-adult DEGS, although a proportion of pharate-adult DEGs were separately grouped. Pw: Newly-ecdysed-pupae; Pp: Pupae-in-apolysis; Pbl: pharate-adults. Red and dark-green horizontal bars represent the functional categories in the comparison between Pw and Pbl phases. Purple and light-green horizontal bars represent the functional categories in the comparison between Pp and Pbl phases.

The GO results for the enriched Biological Process, Molecular Function, and Cellular Component categories are shown in Figure 4. DEGs from newly-ecdysed-pupae and pupae-in-apolysis were grouped in the same Biological Process categories (cellular macromolecule metabolic process; cellular metabolic process involving nucleobases, nucleosides, nucleotides and nucleic acids; macromolecule biosynthetic process; and gene expression including the production of transcripts, translation into proteins, and protein processing events) and Molecular Function (protein binding, nucleic acid binding, and nucleotide binding). By contrast, pharate-adult DEGs were grouped in distinct Biological Process categories (phosphorus metabolic process, electron transport chain, and generation of precursor metabolites and energy), and Molecular Function (structural constituent of cuticle, oxidoreductase activity, substrate specific transporter activity, transmembrane transporter activity, and structural constituent of ribosome) (Figure 4A,B).

Several of the Cellular Component categories were shared by pupal and pharate-adult DEGS, although some of them (membrane, membrane part, organelle membrane, and organelle envelope) were exclusively assigned to pharate-adult DEGs (Figure 4C).

Therefore, GO analysis clearly separated most of the pupal and pharate-adult DEGs into distinct functional categories.

### Differentially expressed CP genes

**Figure 5:**
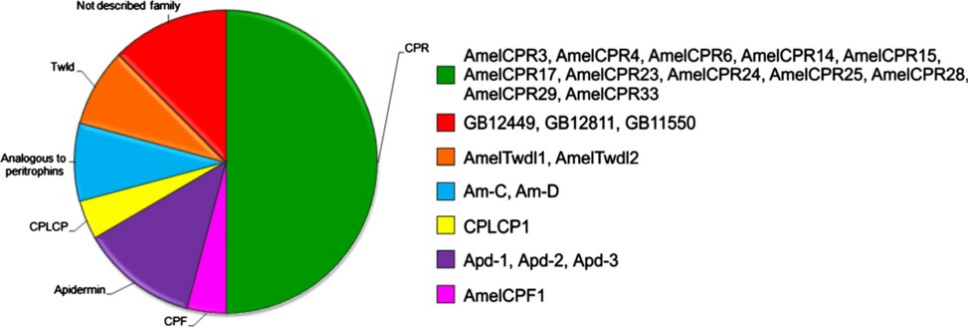
**CP genes differentially expressed in the thoracic dorsum.** Families of CP genes differentially expressed in the thoracic dorsum. The microarray analysis revealed genes encoding members of CPR, CPF, Apidermin, CPLCP, Analogous-to-Peritrophins, and Tweedle (Twdl) families, in addition to putative CP genes not assigned to any family (GB12449, GB12811, GB11550). Genes in each family are listed at the right.

Within the DEGs, we found 24 genes encoding CPs. Figure 5 shows the classes of differentially expressed CP genes detected in the microarrays. The CPR genes (*AmelCPR 3*, *4*, *6*, *14*, *15*, *17*, *23*, *24*, *25*, *28*, *29* and *33*), which encode proteins containing the chitin-binding R&R Consensus [13], were the most abundant among the differentially expressed CP genes (50%). The genes encoding Apidermins (Apd-1, Apd-2, and Apd-3), CPF and CPLCP1 proteins, the Analogous-to-Peritrophins proteins Am-C and Am-D, and the Tweedle1 and Tweedle2 proteins accounted for the remaining differentially expressed CP genes. Another three sequences, GB12449, GB12811 and GB11550, were included in this list of CPs (further details are in the next section).

The structures of the differentially expressed CP genes were determined *in silico* by mapping them to the annotated honeybee genome using Artemis software (Release 7, The Sanger Institute [42]) (Additional file 5: Figure S1). The differentially expressed CP genes comprised 2 exons (8 genes), 3 exons (12 genes), or 4–5 exons (4 genes). The first exon is relatively small in 14 of the differentially expressed CP genes. Schematic representations of the clustered CP genes are shown in Additional file 5: Figure S1, and the characteristics of the proteins encoded by the differentially expressed CP genes are described in Additional file 6: Table S5. All 24 CPs contain an N-terminal signal peptide, consistent with their secretion by the epidermis to integrate the cuticle. A low molecular mass was a common feature shared with CPs from other insects. Of the 24 mature honeybee CPs, 21 (87.5%) have a molecular mass of less than 30 kDa. The smallest CP is 6.1 kDa, and the largest is 40.4 kDa. The R&R Consensus-type was identified using hmmfind software, available at the cuticleDB web site [http://bioinformatics2.biol.uoa.gr/cuticleDB/index.jsp], using default parameters. Among the 12 CPR genes identified in the arrays, six contain the RR-1 Consensus sequence, and five have the RR-2 Consensus sequence. We could not determine whether the CPR gene *AmelCPR33* has the RR-1, RR-2 or RR-3 Consensus sequence.

**Figure 6:**
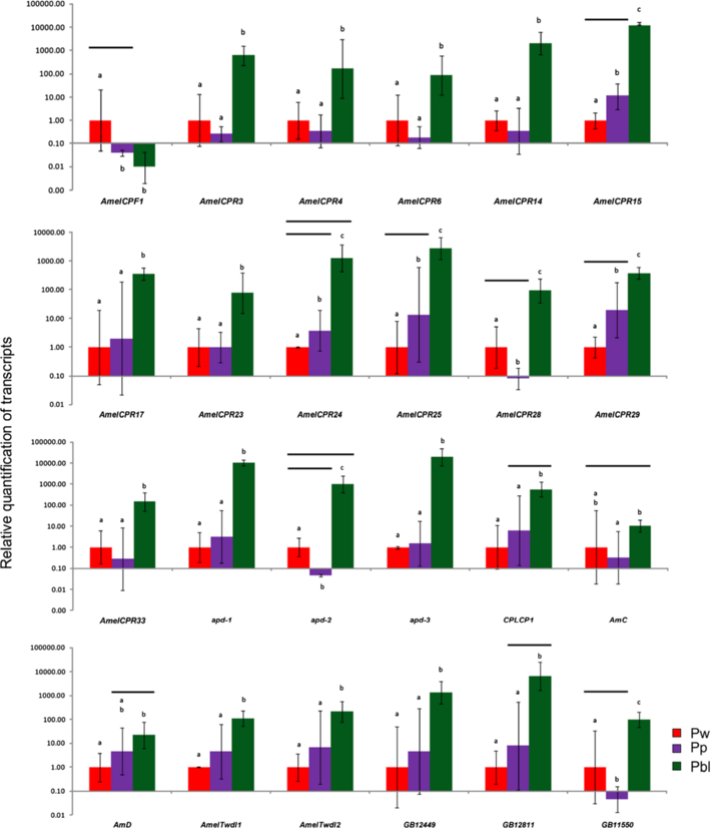
**RT-qPCR for quantification of CP transcripts.** Relative quantification (RT-qPCR) of transcripts encoding CPs in the thoracic dorsum of newly-ecdysed pupae (Pw), pupae-in-apolysis (Pp), and pharate-adults (Pbl). Overall, the RT-qPCR data are in agreement with the microarray data, except for the comparisons marked by horizontal lines above the graph columns.

The expression (transcript levels) of each of the 24 CP genes was quantified by RT-qPCR using specific primers and thoracic dorsum samples from newly-ecdysed-pupae, pupae-in-apolysis, and pharate-adults. The position of the primers in each predicted gene sequence is illustrated in Additional file 5: Figure S1. Seventy-two comparisons were performed in triplicate with independent samples. The RT-qPCR results agreed with the microarray data in 58 of these comparisons, indicating an accuracy of 80.5%. Except for the gene *AmelCPF1* (the unique member of the CPF family revealed in the microarray analysis), all the other 23 CP genes were upregulated in pharate-adults, when the adult cuticle is being deposited (Figure 6).

### GB12449 and GB12811 genes are expressed in the epidermis

The protein FASTA sequence of each DEG was obtained from Amel_prerelease2_OGS_pep data bank (honeybee genome, version 4.0) using GBs as accession numbers. These sequences were analyzed by BLASTP using the non-redundant database (NCBI). The first 10 BLAST hits were searched for the terms “cuticle”, “exoskeleton”, and “cuticular”, which could indicate similarity with described CPs. The predicted proteins encoded by GB12449, GB12811 and GB11550 could not be unequivocally included in any of the 12 previously-defined CP classes [8].

**Figure 7:**
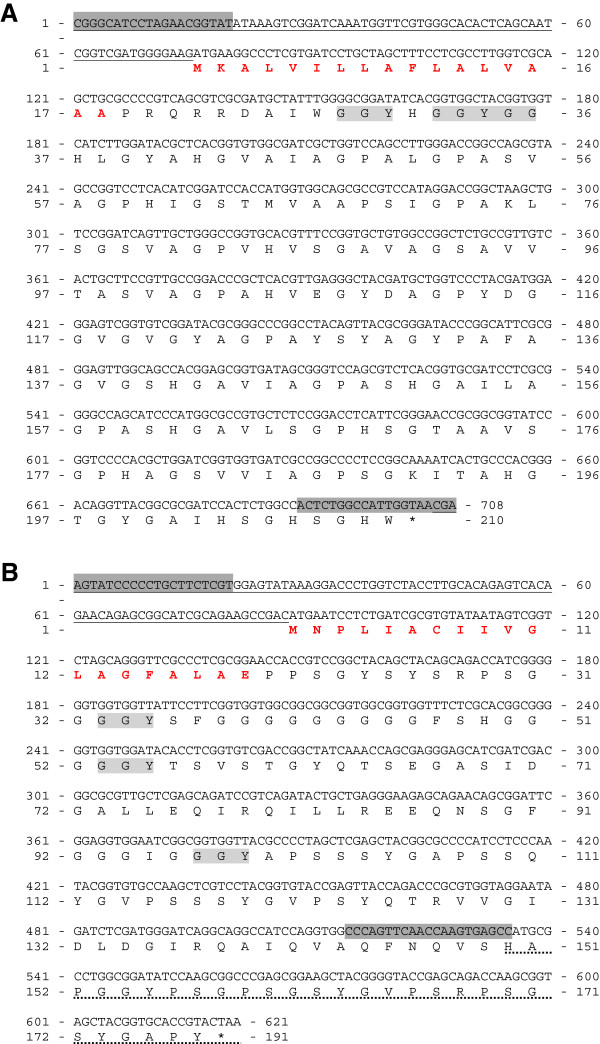
**GB12449 (JX456099) and GB12811 (JX456100) cDNA sequences.** The genes **(A)** GB12449 [GenBank:JX456099] and **(B)** GB12811 [GenBank:JX456100] were validated by sequencing their respective cDNAs. The nucleotides and the predicted amino acid sequences are shown. The 5′UTR regions are underlined. The primers used for sequencing the genes are marked in dark-gray. Signal peptide is in red letters. Conserved motifs are shown in light-gray. Asterisk indicates stop codon. The non-sequenced portion of GB12811cDNA is marked by a dashed line.

Sequencing of the corresponding cDNAs validated the sequences of GB12449 and GB12811 (Figure 7). Both have features that may qualify them as encoding CPs: (1) abundance of glycine residues (GB12449 and GB12811 sequences have 20.9% and 22.5% glycine, respectively); (2) presence of GGYGG and/or GGY motifs, typical of glycine-rich CPs; (3) presence of an N-terminal signal peptide; (4) absence of cysteine residues in the mature protein (GB12811 has a cysteine residue in the signal peptide).

**Figure 8:**
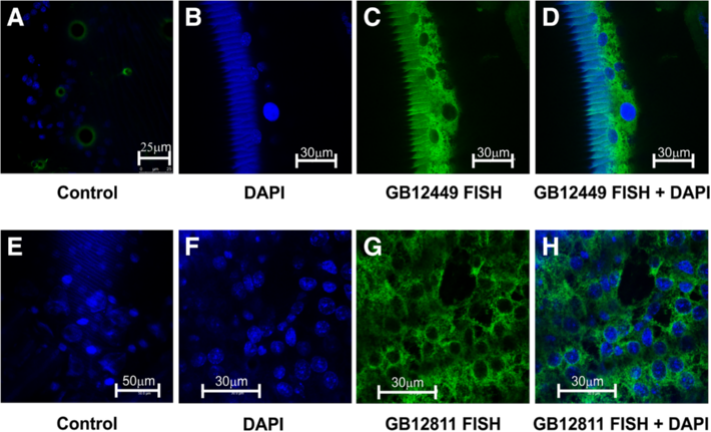
**FISH for localization of putative CP transcripts in epidermis.** Fluorescent *in situ* hybridization and confocal microscopy showing the presence of GB12449 [GenBank: JX456099] and GB12811 [GenBank:JX456100] transcripts (green) in the epidermis of pharate-adult bees. **(A**, **E)** Controls using the sense probes labeled with Alexa Fluor 555® (Invitrogen) and DAPI to stain cell nuclei; **(B**, **F)** DAPI-stained cell nuclei (blue); **(C**, **G)** GB12449 and GB12811 antisense probes labeled with Alexa Fluor 555® (Invitrogen); **(D**, **H)** Merged B + C and F + G images. **A**, **E**-**H**: monolayer of epidermal cells in focal planes. **B**-**D**: cross sections of the integument showing the cuticle at the left and the epidermis at the right. Setal sockets **(**green rings in **A)** and the cuticle **(**seen in **B**-**D)** are self-fluorescent.

Using *in situ* hybridization, GB12449 and GB12811 transcripts were localized in the cytoplasm of epidermal cells in the thoracic dorsum of pharate-adults (Figure 8), where these genes were expressed at high levels (see transcript levels in Figure 6). In summary, these genes are expressed in the epidermis and possibly encode proteins involved in cuticle formation.

The protein encoded by GB11550 was identified in integument extracts from honeybee pupae and pharate-adults using 2-dimensional electrophoresis (data not shown). Well-focused protein spots were trypsin-digested, and identified by peptide mass fingerprint using MALDI-TOF mass spectrometry. Mass spectra were analyzed against the NCBI non-redundant protein database using Mascot software for protein identification and characterization. This analysis confirmed the presence of the GB11550 protein in the integument [43].

### Differential expression of genes related to thoracic muscle formation

Among the DEGs, we detected genes that are potentially involved in the differentiation of thoracic muscles from precursor cells. Myoblasts derived from mesenquimal cells are the precursors of myocytes that elongate and fuse to form the adult thoracic muscles [1]. Conventional light microscopy analysis of the thoracic dorsum of pupae-in-apolysis (Figure 1) showed a mass of myocytes subjacent to the integument and surrounded by fat cells. The myocytes differentiate into striated muscle fibers that contact specific regions of the epidermis. Functioning as tendons, the integument infolds (i.e., apodemes) and specific points in the superficial epidermis serve as attachment sites for the striated muscles. Muscle attachment to the epidermis starts in early pharate-adults (at the Pb phase, which immediately precedes the Pbl phase). In the thoracic dorsum of the Pbl phase, the musculature is already in close contact with the integument (Figure 1).

Among the 36 genes identified as “muscle genes” in the DEG list, 28 were upregulated during thoracic musculature formation (Additional file 3: Table S3; Additional file 4: Table S4). These genes are potentially involved in functions such as structural constituents of muscle fibers, muscle tendon junction, muscle attachment, adult somatic muscle development and sarcomere organization.

Before the onset of muscle fibers formation from precursors, the thoracic dorsum is filled with fat body cells (Figure 1: see thoracic cross section of Pp pupa-in-apolysis). As development proceeds, the fat body cells are gradually replaced by the thoracic musculature (Figure 1, see thoracic cross section of Pb and Pbl pharate-adults). The hexamerin genes highly expressed in the fat body, *hex 70a* (GB30362), *hex 70b* (GB10869), *hex 70c* (GB13613) and *hex 110* (GB14361), were revealed in the microarray analysis of pupal thoracic dorsum. Their transcript levels were reduced in the subsequent developmental phases, when the fat body cells become scant or disappear from the thoracic dorsum (see Additional file 3: Table S3; Additional file 4: Table S4).

In summary, the developmental morphology of the thoracic dorsum was in agreement with the expression of fat body and muscle-related genes in the microarray data.

### Regulatory motifs shared by the DEGs

**Figure 9:**
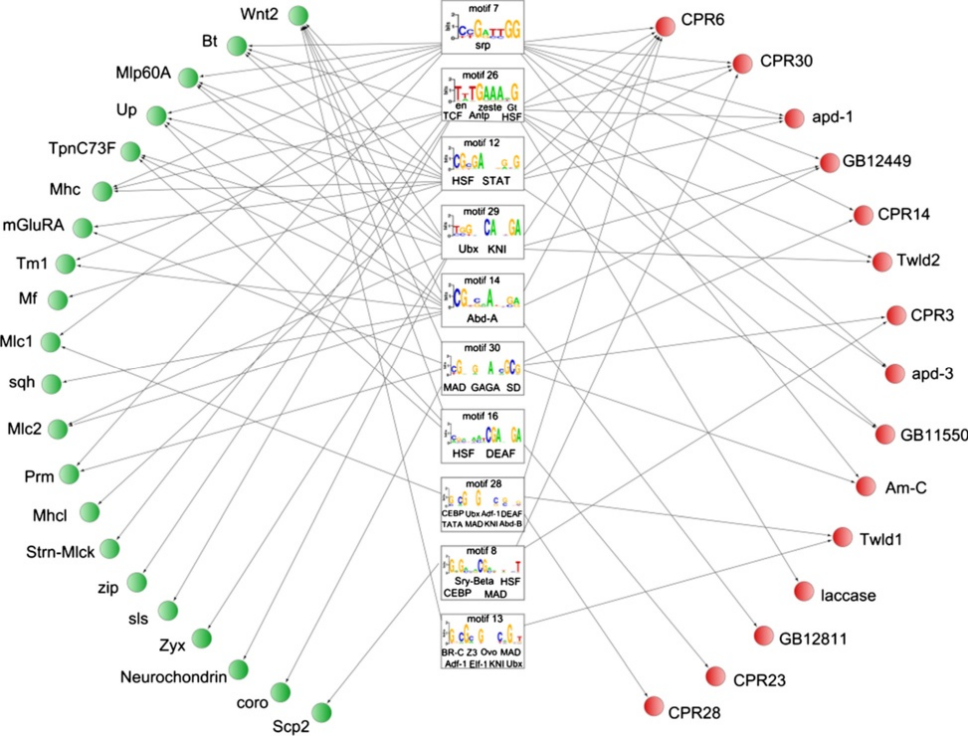
**Proposed regulatory network in exoskeleton construction.** Potential regulatory network acting in the thoracic dorsum based on shared overrepresented motifs in the UCR regions of CP- and muscle-related genes. CP- and muscle-related genes integrating the network were all up regulated during adult exoskeleton formation. Rectangles show transcription factors and the target sequences (motif logos) in the UCR regions (see Additional file 7: Table S6). Red and green circles represent the CP- and muscle-related genes, respectively. The arrows indicate the occurrence of motifs in the UCRs of target genes. See Additional file 6: Table S5 for GB accession numbers and other information on these CP genes. Muscle-related genes: Wnt2 (GB16243), Bt (GB11358), Mlp60A (GB19606), Up (GB16881), TpnC73F (GB10545), Mhc (GB30329), mGluRA (GB18621), Tm1 (GB30512), Mf (GB12239), Mlc1 (GB16215), sqh (GB17693), Mlc2 (GB13399), Prm (GB30222), Mhcl (GB12303), Strn-Mlck (GB16909), zip (GB18651), sls (GB14642), Zyx (GB15339), Neurochondrin (GB14786), coro (GB20076), Scp2 (GB15916). See Additional file 3: Table S3 and Additional file 4: Table S4 for information on identity and potential function of muscle-related genes.

Thirty motifs resembling previously identified transcription factor binding sites (with greater than 70% similarity) were discovered in the UCRs of the honeybee DEGs (Additional file 7: Table S6). Ten of these *cis*-regulatory motifs were shared by several of the upregulated CP- and muscle-related genes. The regulatory network shown in Figure 9 organizes all this information and suggested that CP- and muscle-related genes are co-regulated in the developing thoracic dorsum.

Motif_7 and motif_26 showed greater than 80% similarity with binding sites for known transcription factors. Motif_7 is 83% similar to the Serpent (SRP) binding site. SRP is a GATA-like transcription factor involved in the specification of mesoderm-derived tissues in *D. melanogaster*, including the thoracic musculature. Motif_26 showed 87% similarity to the Antennapedia (Antp) binding site motif, 85% similarity to the Engrailed (En) and Zeste (Z) binding site motifs, 82% similarity to the Giant (Gt) binding site and 88% similarity to the T-cell-factor (TCF) binding site. Importantly, Antp, En, Z, and TCF are all expressed in the precursors of the thoracic dorsum, i.e., the wing imaginal discs (references in Discussion, part 2).

We also found binding sites sharing more than 70% similarity with Broad-Complex (BR-C Z1, BR-C Z2 and BR-C Z3 isoforms) response elements (Additional file 7: Table S6). Broad-Complex isoforms are ecdysone signal-related transcription factors reported to be important during adult cuticle differentiation [44]. The presence of a BR-C Z3-like binding site (motif 13) in the regulatory region of genes encoding a CP (Tweedle1) and a muscle-related protein (Wnt2) (Figure 9) suggests direct co-regulation by ecdysone during thoracic dorsum formation.

There is experimental evidence that the FTZ-F1 transcription factor regulates the transcription of CP genes in *B. mori* and *D. melanogaster* (reviewed in [45]); therefore, we also searched for FTZ-F1 binding sites in the promoter region of the upregulated CP- and muscle-related gene set shown in Figure 9. Except for the occurrence of one FTZ-F1-like binding site in the Am-C gene (GB13298; Figure 9), which is very similar (over 80%) to the consensus sequence, the presence of FTZ-F1-like binding sites was not supported by any of three enrichment scores used in our computational analysis (pipeline designed for cis-regulatory motifs discovery; Church >0.05, ROC-AUC <0.5 and Enrichment score > 0.05).

## Discussion

### Differential expression of genes involved in thoracic dorsum formation during the ecdysteroid-regulated pupal-to-adult molt

Our microarray data underscored the intense changes in gene expression in the thoracic dorsum during the pupal-to-adult molt, and revealed a set of genes involved in adult cuticle formation. GO analysis clearly grouped the pupal and pharate-adult DEGs into distinct Biological Process and Molecular Function categories, and revealed the complexity of functions needed for the relatively rapid process of definitive exoskeleton formation.

The most represented GO categories among the pupal DEGs were related to DNA replication, transcription, mRNA splicing and polyadenylation, tRNA metabolic process, rRNA processing, translation, protein folding and Golgi organization. This is in agreement with DNA, RNA and protein syntheses occurring in the epidermis for cell division or increase in size [46]. Other GO categories associated with pupal DEGs (mitotic spindle organization and elongation, cyclin catabolic process, mitotic anaphase-promoting complex activity, regulation of mitotic metaphase/anaphase transition, transcription involved in G2/M phase of mitotic cell cycle and regulation of cell cycle) suggest roles in cell division. Such activities precede, and are usually correlated with, the increase in ecdysteroid titer and apolysis. Furthermore, secretion and membrane trafficking apparently occurs in response to ecdysone, which has been reported to induce Golgi formation (reviewed in [47]).

By contrast, the most represented GO categories among pharate-adult DEGs were mainly related to mitochondrial activity and energy generation (mitochondrial electron transport, oxidative phosphorylation, and ATP synthesis coupled electron transport). The GO categories including structural constituent of the cuticle, peroxidase activity, oxidoreductase activity and transmembrane and protein transport, could be intuitively connected to adult cuticle deposition and differentiation.

The most prominent epidermal products involved in cuticle formation are the structural CPs. Based on sequence motifs, the CPs identified to date in insects and other arthropods were tentatively classified into 12 different families [8]. Only six of the 12 CP families have been predicted in the honeybee genome. Proteins in these six families are encoded by 47 CP genes, of which 32 have the R&R Consensus sequence characteristic of the CPR family, three encode proteins from the CPF/CPF-like families [48], two encode Tweedle proteins [49], two encode CPLCP proteins (Cuticular Protein of Low Complexity, Proline-rich [50]), three encode Apidermins [14] and five encode CPAP3 family proteins (Cuticular Proteins Analogous to Peritrophins having 3 ChtBD2 chitin-binding domains) [51].

The whole-genome oligonucleotide array analysis revealed 24 CP genes, including six that were previously validated [12,14,15]. All but one (*AmelCPF1*) of these genes was upregulated during the synthesis and deposition of the adult cuticle. Twelve of them encode CPR proteins. Eight genes encode CPs from the Apidermin, CPLCP, Analogous-to-Peritrophin and Tweedle families. The other three upregulated genes (GB12449, GB12811 and GB11550) could not be categorized into a CP family. However, GB12449 and GB12811 sequences showed motifs and features that have been frequently used to identify putative CP genes in insect genomes. Furthermore, *in situ* hybridization experiments showed that GB12449 and GB12811 transcripts are abundant in the epidermis during adult cuticle deposition, suggesting that their products are structural CPs. The GB11550 translation product was identified in the integument by 2-dimensional electrophoresis and mass spectrometry, and was found in higher levels in pharate-adults than in pupae [43]. The upregulation of these 23 CP genes in pharate-adults, as verified in the microarray data, was confirmed by RT-qPCR transcript quantification. All these genes are induced after apolysis, i.e., after the ecdysteroid pulse, coinciding with adult cuticle deposition.

As revealed by microarray hybridization and RT-qPCR analyses, *AmelCPF1* did not fit the typical pattern of CP gene regulation because its expression is higher in the integument of newly-ecdysed-pupae than in pupae-in-apolysis and pharate-adults. This suggests that *AmelCPF1* is downregulated during apolysis by the ecdysteroid peak. This gene is apparently not involved in adult thoracic cuticle formation, although it may have a role in structuring the pupal cuticle. Using stringent criteria for characterizing stage-specific CPR family genes in *Anopheles gambiae*, Togawa *et al*. [10] identified genes that appeared to be specific to a single developmental stage, namely, the larval, pupal, or adult stages. The investigation of *AmelCPF1* expression in other developmental stages, or instars, may help to elucidate its stage-specific character.

The RT-qPCR findings highlighted the transient and statistically significant downregulation of *AmelCPR28, apd-2* and *GB11550* during apolysis (see Figure 6), when the ecdysteroid titer is maximal. Several CP genes are downregulated *in vivo* by the high ecdysteroid titer that induces apolysis, or *in vitro* by a high ecdysteroid concentration; expression is then recovered following the hormone peak, or after its removal from the incubation medium (reviewed in [45]). The ecdysteroid peak seems important for reprogramming these genes, and other genes involved in the molting process, for later expression. This type of ecdysteroid-modulated expression was previously demonstrated for *AmelCPR14*[12], *AmelTwdl1* and *AmelTwdl2*[15]. The microarray and RT-qPCR findings have extended the number of CP genes that are potentially upregulated by the ecdysteroid pulse for construction of the adult exoskeleton.

The CPR genes upregulated in the thoracic dorsum of pharate-adults encode proteins containing RR-1 (6 genes) or RR-2 (5 genes) Consensus sequences, implying that both contribute to the structure of the hard (rigid) thoracic cuticle. This finding contrasts with earlier studies connecting RR-1 to soft (flexible) cuticles and RR-2 to hard cuticles. This is a subject not yet fully resolved, because studies on cuticle genes and proteins have been mainly made with whole body extracts. Andersen [52] suggested that RR-2 proteins are part of the exocuticle deposited during the pharate stage, whereas RR-1 proteins integrate the post-ecdysial endocuticle. However, this hypothesis has not yet been unequivocally validated [8,10].

The differential expression of genes encoding enzymes such as prophenoloxidase, tyrosine hydroxylase, and dopa-decarboxylase, which catalyze reactions leading to cuticle melanization and sclerotization, was not detected by the microarray analysis. We previously used RT-qPCR to investigate the expression of these genes in the honeybee integument [16,53]; there were significant increases in transcript levels in pharate-adults (Pbm and Pbd phases) older than those used in the current study (Pbl phase) (see the developmental phases in Figure 1). Consequently, the genes encoding these enzymes were not detected among the DEGs in our microarray analysis. However, other genes potentially involved in cuticle tanning, such as those encoding a laccase (*Amlac2*) and five peroxidases, were upregulated in pharate-adults. *Amlac2* encodes a laccase involved in the cross-linking of CPs and quinones for cuticle sclerotization [54]. Two of the peroxidase genes, GB13459 and GB10387, displayed 20% and 32% similarity (ClustalW 2.1 score), respectively, at the protein level with the translation product of a previously described cuticular peroxidase gene, *Ampxd* [GenBank:ADE45321.2] [15]. These Animal Heme Domain-containing peroxidases catalyze oxidative reactions, and may play a role in the oxidation of catechols to quinones, which leads to cuticle sclerotization [55,56].

The other peroxidase genes included a glutathione peroxidase (GB14138) and two peroxiredoxins (GB10498 and GB10972), also known as thioredoxin peroxidases. The honeybee glutathione peroxidase gene was previously included in a study on the evolution of this gene family in invertebrates [57]. Glutathione peroxidases and peroxiredoxins have been characterized in numerous taxa as important antioxidants. Both proteins were identified in the cuticle of parasitic nematodes, possibly for protection against reactive oxygen species generated by the defense system of the host (reviewed in [58]).

Like the CP genes and peroxidase genes identified in the microarray analysis, *Amlac2* and *Ampxd* displayed increased expression in pharate-adults, following the ecdysteroid pulse.

### CP- and muscle-related genes in the thoracic dorsum may share common regulatory motifs

Studies in *Drosophila* have shown that the notum epidermis and subjacent muscle fibers share the same precursors, i.e., both develop from the wing imaginal discs [23]). The epithelial cells of the discs are ectodermal in origin and differentiate into the notum and wing epidermis. Mesodermal adepithelial cells associated with the wing imaginal discs give rise to myoblast precursors of flight muscles [59]. Ten of the 30 potential binding sites for known transcription factors in the TRANSFAC database were shared by upregulated CP- and muscle-related genes (see Figure 9). Thus, these genes may be co-regulated during the differentiation of the thoracic exoskeleton. Two of these motifs, motif_7 and motif_26 (see Figure 9), are noteworthy because they are more than 80% similar to binding site motifs for known transcription factors.

Motif_7 is similar to the SRP transcription factor binding site. SRP is important for differentiation of mesoderm-derived tissues, such as the fat body and muscle precursors, in *D. melanogaster* embryos [60], and may downregulate the expression of a gene essential for myogenesis: *Mef2* (Myocyte-specific enhancer factor 2) [61,62].

Motif_26 is highly similar to binding sites for transcription factors active in developing wing imaginal discs in *Drosophila*, such as Antp, Z, En, and TCF. *Antp* is expressed in the presumptive notum (thoracic dorsum) region [63]. During metamorphosis, *z* is expressed in all imaginal disc derivatives and in the thoracic musculature of pharate-adults [64]. *en* plays a major role in the territorial subdivision of the imaginal wing discs for wing and thoracic dorsum (notum) definition (reviewed in [65]). dTCF (also called Pangolin) mediates the Wingless (Wg) signal transduction pathway in the wing imaginal discs. Like *en*, Wg is a player in wing disc territorial subdivision, and is involved in intercellular signaling that specifies the wing and notum primordia [66,67]. It is likely that factors active in the precursors (wing imaginal discs) remain active in the tissues derived from them (thoracic dorsum). This is an inference awaiting functional analysis.

Several of the potential transcription factor binding sites identified in the honeybee DEGs were previously found in *A. gambiae* CPR genes [10], such as Zen, Gt, Tll, Eve, Ubx, Abd-B, Twi, Z, and Broad-Complex isoforms (BR-C Z1, Z2, and Z3), the ecdysone signaling-related transcription factors. There is experimental evidence that Broad-Complex proteins regulate the transcription of CP genes in *B. mori*[68,69].

The presence of binding sites for BR-C isoforms suggests that ecdysone directly regulates some DEGs during thoracic dorsum formation. As we previously demonstrated, the expression of the CP gene *AmelTwdl1* is modulated by the ecdysteroid titer [15]. The discovery of a potential BR-C Z3-like binding site motif in the *AmelTwdl* UCR (motif_13 in Figure 9) is consistent with this finding. In *D. melanogaster*, the attachment of thoracic muscles to the dorsal epidermis requires BR-C Z1 function. It was proposed that *BR-C Z1* is induced by ecdysteroids in the thoracic wall and regulates target genes involved in tendon cell maturation [70]. Ecdysteroids also plays a key role in adult muscle development [71]. The muscle-related gene *Wnt2* has a potential BR-C Z3-like binding site motif (motif_13 in Figure 9), suggesting its regulation by ecdysteroids. In *Drosophila Wnt2* mutants, flight muscles are lost or fail to attach to their epidermal targets during adult thoracic muscle formation [72].

In addition to *tweedle 1* and *Wnt2*, other upregulated CP- and muscle-related DEGs contain potential binding sites for BR-C isoforms. Motif_25, which is similar to the BR-C Z1 binding site, was identified in the CP gene encoding an apidermin, *apd-2*; motif_9, a possible binding site for BR-C Z2/Z3, was detected in the muscle-related gene *myofilin*. Similarly, putative binding sites for BR-C Z2 (motif_15) were identified in genes encoding a peroxidase and a peroxiredoxin. Both enzymes have been associated with cuticle pigmentation and sclerotization [15,56], which are regulated by ecdysteroids [73,74]. These motifs were omitted from the regulatory network (Figure 9) because they were identified in CP- *or* muscle-related genes, and the regulatory network was constructed exclusively with those motifs detected in both CP- *and* muscle-related genes.

Functional analysis has shown that FTZ-F1 regulates the transcription of CP genes in *B. mori* and *D. melanogaster* (reviewed in [45]). Although FTZ-F1 binding sites are not enriched in the honeybee CP genes, the occurrence of a putative site in the Am-C gene (GB13298; Figure 9) may suggest this cuticular gene is regulated by FTZ-F1.

Our bioinformatics approach only hints at the complex regulatory network supposedly involved in thoracic exoskeleton development. By integrating our data in a gene network (Figure 9) we provide a hypothetical framework, which can be the basis for further functional analyses.

## Conclusions

Our data broadens our understanding on thoracic dorsum formation, at the molecular level, during the pupal-to-adult molt. Gene expression changes associated with formation of the hard thoracic dorsum in the honeybee were demonstrated using genome-wide cDNA microarray analysis and RT-qPCR. Of the 1,253 DEGs, 547 were upregulated in the thoracic dorsum following apolysis, implying induction by the ecdysteroid pulse that triggers cuticle deposition. CP genes potentially encoding proteins of the CPR, Tweedle, Apidermin, CPF, CPLCP1, and Analogous-to-Peritrophins families were associated with thoracic dorsum formation. Three other genes, which do not belong to any known CP families, are novel CP candidate genes. In support of this hypothesis, fluorescent *in situ* hybridization revealed abundant expression of two of these novel genes in the pharate-adult epidermis, which is actively engaged in adult exoskeleton synthesis. Several potential binding sites for biologically relevant transcription factors were shared by upregulated CP- and muscle-related genes, suggesting co-regulation for coordinated development of thoracic exoskeleton and subjacent striated musculature. Further functional analysis of these *cis*-elements will be needed to test this hypothesis.

## Abbreviations

Abd-B: Abdominal-B; Antp: Antennapedia; ATP: Adenosine triphosphate; BR-C Z1, BR-C Z2, BR-C Z3: Broad-Complex isoforms; cDNA: Complementary DNA; CP: Cuticular protein; CPAP3: Cuticular protein analogous to peritrophins having 3 ChtBD2 chitin-binding domains; CPF: Cuticular protein containing a 41–44 aminoacids conserved sequence; CPLCP: Cuticular protein of low complexity- proline-rich; CPR: Cuticular protein containing a Rebers and Riddiford Consensus; Ct: Threshold cycle; DAPI: Diamidino-2-phenylindole; DAVID: The data base for annotation, visualization and integrated discovery; DMSO: Dimethyl sulfoxide; DNAse: Deoxyribonuclease; DEG: Differentially expressed gene; DNA: Deoxyribonucleic acid; dTCF: Drosophila T-cell factor; En: Engrailed; Eve: Even-skipped; FDR: False discovery rate; FTZ-F1: Fushi-tarazu factor 1; GEO: Gene expression omnibus database; GO: Gene ontology; Gt: Giant; HS: Hybridization solution; mRNA: Messenger ribonucleic acid; Mef2: Myocyte-specific enhancer factor 2; MIAME: Minimum information about a microarray experiment; NCBI: National center for biotechnology information; OGS: Official gene set sequences; ORF: Open reading frame; Pb: Pbl, Pbm, Pbd, Successive pharate adult phases in honeybee development; PBS: Phosphate buffered saline; PCR: Polymerase chain reaction; Pp: Honeybee pupae-in-apolysis; PSSM: Position-specific scoring matrix; Ptw: Phosphate buffered saline containing Tween-20; Pw: Honeybee newly-ecdysed pupae; rRNA: Ribosomal ribonucleic acid; RNA: Ribonucleic acid; RR-1: RR-2, R&R Consensus types; RT: Reverse transcription; RT-qPCR: Real-time reverse transcription polymerase chain reaction; SDS: Sodium dodecyl sulfate; SRP: Serpent transcription factor; SSC: Saline sodium citrate buffer; TCF: T-cell factor; Tll: Tailless; tRNA: Transfer ribonucleic acid; Twi: Twist; Ubx: Ultrabithorax; UCR: Upstream control region; UV: Ultraviolet light; Wg: Wingless; Z: Zeste; Zen: Zerknüllt.

## Competing interests

The authors declare that they have no competing interests.

## Authors’ contributions

MPMS and ARB performed the microarray analysis. ACQS analyzed the microarray data. MPMS performed the RT-qPCR analysis, fluorescent *in situ* hybridization, and prepared all figures and additional files. ACS and FCPF performed the motif analyses and constructed the regulatory network. LLC provided the integument histological sections. MMGB designed the study and wrote the manuscript. All authors read and approved the final manuscript.

## Supplementary Material

Additional file 1: Table S1 Primers used for the RT-qPCR analysis. Click here for file

Additional file 2: Table S2 Primers used for Fluorescent *in situ* hybridizations and sequencing. Click here for file

Additional file 3: Table S3 Genes differentially expressed in the comparison between newly-ecdysed-pupae (Pw phase) and pharate-adults (Pbl phase). Click here for file

Additional file 4: Table S4 Genes differentially expressed in the comparison between pupae-in-apolysis (Pp phase) and pharate-adults (Pbl phase). Click here for file

Additional file 5: Figure S1 Schematic representation of the structure of the differentially expressed CP genes. Click here for file

Additional file 6: Table S5 Differentially expressed CP genes and characteristics of their deduced proteins. Click here for file

Additional file 7: Table S6 Thirty motifs (cis-elements) sharing more than 70% similarity with binding sites for known transcription factors. Click here for file
