# Supplementary material for: Genes involved in thoracic exoskeleton formation during the pupal-to-adult molt in a social insect model, Apis mellifera
Source: BMC Genomics. 2013 Aug 28;14:576. doi: 10.1186/1471-2164-14-576 (PMC3766229; doi:10.1186/1471-2164-14-576)

**Figure S1- Schematic representation of the structure of the differentially expressed CP genes. (A)** Schematic representation of the structure of the differentially expressed cuticle protein genes. The long arrow below representations of gene structure indicates the direction of transcription. **(B)** Schematic representation of the cuticle protein genes arranged in clusters in the following linkage groups of the honey bee genome (version 4.5): Group2.7, Group4.7 and GroupUn41 (the apidermin gene cluster was described by **Kucharski *et al*. [14],** based on the honey bee genome, version 2.0). The genes and transcription direction are represented by large arrows. In **(A)** and **(B)** the exons and introns are indicated by boxes and lines, respectively, and the number of nucleotides is shown; black arrows indicate primers used in RT-qPCR, while small grey arrows indicate primers used in *in situ* hybridizations. The clear portion of the 5’ boxes in the GB12449 and GB12811 genes represent the UCR region where primers were designed for sequencing these genes (as indicated by the small black arrows).


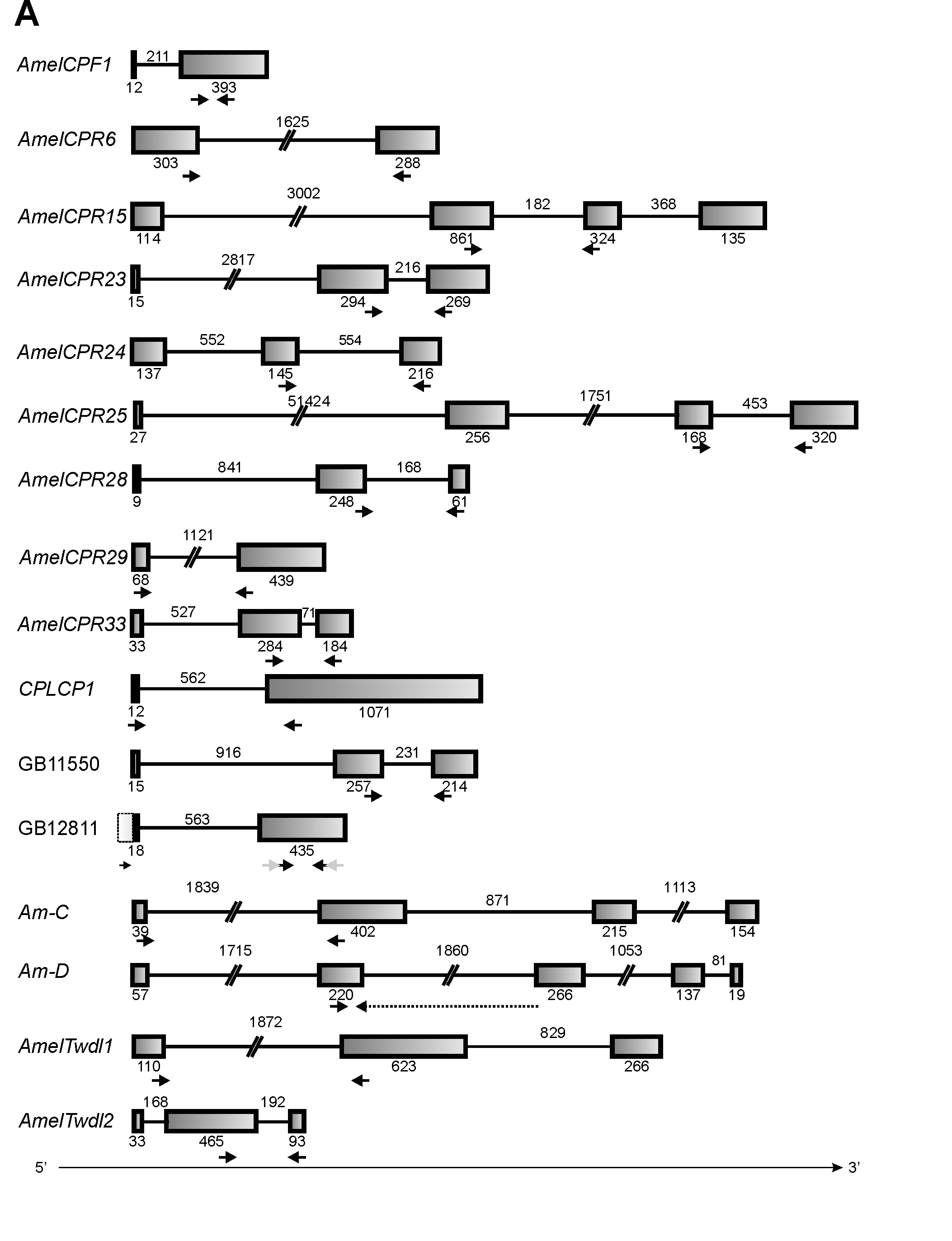

Supplement: Additional file 5: Figure S1 — Schematic representation of the structure of the differentially expressed CP genes. [file 1471-2164-14-576-S5.docx]
